# Social cognitive elements of mental illness stigma among healthcare professionals currently working in general hospitals: A cross‐sectional study from Jordan

**DOI:** 10.1002/nop2.1953

**Published:** 2023-07-29

**Authors:** Heyam F. Dalky, Malek Alnajar, Ala'a F. Dalky, Naser Mahmoud, Mohammad Al‐Ma'ani, Sultan Mosleh, Ayman M. Hamdan‐Mansour

**Affiliations:** ^1^ Community/Mental Health Nursing Department, Faculty of Nursing Jordan University of Science & Technology Irbid Jordan; ^2^ Graduate Research and Teaching Assistant College of Nursing University of Utah Salt Lake City Utah USA; ^3^ Department of Health Management and Policy, Faculty of Medicine Jordan University of Science and Technology Irbid Jordan; ^4^ Acute Inpatient Psychiatry Unit Al‐Rashid Hospital Center Amman Jordan; ^5^ Health Science University of Fujairah Fujairah United Arab Emirates; ^6^ Health Sciences Department‐Nursing Program Higher Colleges of Technology Al‐Fujairah United Arab Emirates; ^7^ School of Nursing The University of Jordan Amman Jordan

**Keywords:** general hospital, healthcare professional, Jordan, mental illness, stigma

## Abstract

**Aim:**

To assess the social cognitive elements of the stigma of mental illness (knowledge, attitudes and behaviours) among healthcare professionals (HCPs) in Jordan.

**Design:**

A cross‐sectional descriptive design.

**Methods:**

A total of 206 HCPs were conveniently recruited from general hospitals in Jordan. The mental attitude, knowledge and intended behaviours scales were used to measure stigma elements.

**Results:**

Participants reported a moderate level of knowledge, a moderate negative attitude and a moderate or not greater interest to deal with people with mental health illnesses. The bivariate correlation revealed a negative significant correlation between HCPs' knowledge and attitude, indicating that HCPs with more knowledge significantly have more positive attitude (lower average score) towards those suffering from the illness. A more significant correlation was found between HCPs' knowledge and behaviour. The HCPs who had more knowledge were holding more interest and willingness towards dealing with persons with mental illness.

**Patient or Public Contribution:**

Negative attitudes among HCPs demand awareness programmes pertaining to the stigma of mental illness to afford higher standards of practice for patients with mental problems.

## 1 INTRODUCTION

Mental illness is one of the extremely stigmatized chronic illnesses. Recently, the perception of stigma that has become apparent is that this stigma does not tackle only general community but also become more prevalent among healthcare professionals (hereafter HCPs) (Dalky et al., 2020; Stuber et al., 2014; Thornicroft et al., 2016). In fact, healthcare professionals reported more stigmatizing attitudes when compared to the general public; particularly the perception of dangerousness (Reavley et al., 2014). Further, stigma of mental illness appears in two main forms. Public stigma refers to the perception of negative stereotypes of people with mental illness by public (Angermeyer & Dietrich, 2006), whereas self‐stigma occurs when persons with mental illnesses concur with and internalize such negative stereotypes (Corrigan et al., 2006; Livingston & Boyd, 2010). Evidence has shown that stigma perceptions among HCPs could vary considering the mental healthcare contexts and personal and public attitudes surrounding mental illnesses.

Studies showed that HCPs working at general hospitals reported poor skills and knowledge of mental health issues resulting in uncertainty and a perception of dangerousness when caring for mentally ill patients (Giandinoto & Edward, 2015). The false or negative perceptions among HCPs that stigmatizes the mentally ill as dangerous, unpredictable, violent, and bizarre may give rise to fearful attitudes. Therefore, HCPs may engage in avoidance behaviours to minimize the perception of dangerousness (Giandinoto & Edward, 2015). Despite HCPs' contact with patients with mental illness and the belief that mental health care is an essential component of holistic patient care, HCPs reported negative attitudes and stereotypes towards patients with mental illness (Gronholm et al., 2017). Such negative attitudes may negatively impact the quality of care provided leading to a poor quality of life of patients (Gronholm et al., 2017). Of further concern is the quality of care and the consequences of negative attitudes on HCPs and services users.

Stigma perpetuated by HCPs against clients with mental illnesses has been conceptualized as an overarching social cognitive term of three components: ignorance (problems of knowledge), prejudice (problems of attitude) and discrimination (problems of behaviour; Thornicroft et al., 2007). Healthcare professionals often hold negative stigmatizing perceptions, attitudes and behaviours towards clients with mental illnesses (Gronholm et al., 2017; Thornicroft et al., 2007).

### 1.1 Stigma associated with mental illness: Perceptions from Jordan

In Jordan, patients with mental illness and their families suffer from the stigma associated with their illness (Dalky et al., 2017; Rayan & Aldaieflih, 2019). Stigma among people with mental illness coupled with negative attitudes and behaviours of HCPs would hinder those people to seek mental health care services (Al Ali et al., 2017; Dalky et al., 2020; Hamdan‐Mansour & Wardam, 2009). This explained why the majority of Jordanian patients tend to use informal or traditional health services instead of formal ones (Al Ali et al., 2017). Stigma does not serve only as a barrier to access and utilize mental health services, but it has a significant impact on patients and their family caregivers in terms of quality of life (Dalky, 2012; Dalky et al., 2017). To the researcher's knowledge, few research studies have been conducted in Jordan to address experiences of stigma among HCPs while caring for patients with mental illness (Dalky et al., 2020; Hamdan‐Mansour & Wardam, 2009). Few if no study has been conducted or targeted hospital‐working HCPs (physicians and nurses). Therefore, this study aimed to assess the levels of social cognitive stigma elements (knowledge, attitudes, and behaviours) among hospital‐working HCPs and to identify factors that correlate with or possibly predict those elements. In this study, hospital‐working healthcare professionals are nurses or physicians who are currently working in general hospitals in Jordan.

### 1.2 Social cognitive theoretical model of stigma

Conceptually, stigma has a long history in social science. Goffman' (1963) book, “Stigma: Notes on the Management of Spoiled Identify,” is seen as a classic example of research into the consequences, nature of and sources of stigma (Link et al., 2004). As a term, “mental illness stigma” is not limited to one definition and varies in meaning and conceptual definitions among scientists (Link et al., 2004). Furthermore, a variety of contexts and populations have been tested using multiple theoretical models.

Three paradigms have been identified in the literature that try to explain stigma: the sociocultural perspective, the motivational perspective and the social cognitive theories (Crocker & Lutsky, 1986). According to the sociocultural perspective, stereotypes and other stigmatizing beliefs and cognitions are functions of the culture; the stigma of mental illness is transmitted from one generation to another through the process of socialization. According to the motivational perspective, prejudice or other certain beliefs about a stigmatized group have individual origins. In addition, in the social cognitive theories, stereotypes are considered normal responses to the cognitive operations.

In this study, the authors utilized Thornicroft et al. (2007) social cognitive theory of stigma to guide the definition and measurement of stigma perception. Accordingly, stigma is defined in terms of knowledge, namely ignorance; attitude, or more exactly prejudice and behaviour, more specifically discrimination (Thornicroft et al., 2007). In effect, stigma manifests because of inaccurate knowledge regarding mental issues, which in turn allows negative attitudes to develop in the individual. This negativity consequently causes unacceptable behaviour, for example discrimination.

This can be further illustrated. False beliefs about the mentally ill, for example, they pose a danger, may result in negative attitudes towards those people. In turn, this results in behavioural response or discrimination. For example, “I will not allow dangerous people to get close to me.” The instruments developed by Thornicroft and colleagues (Evans‐Lacko et al., 2010; Evans‐Lacko et al., 2011; Gabbidon et al., 2013); are being used in this study to assess knowledge, attitudes, and reported or intended behaviours for a sample of HCPs in selected general hospitals in Jordan.

## 2 METHODS

### 2.1 Design and sample

A cross‐sectional descriptive design was used to assess the components of stigma; knowledge, attitudes, and behaviours. The study adhered to the STROBE guidelines (Cuschieri, 2019) for cross‐sectional studies. A convenience sample of 206 HCPs (physicians and nurses) were recruited from general hospitals in Jordan from June 2020 to June 2021. Data collection duration time extended due to the COVID‐19 pandemic and the restrictions it imposed in the healthcare system and working duties. The names of hospitals are kept confidential. The sample size was determined using G‐power analysis 3.0.10 using the Pearson correlation coefficient test with large effect size of 0.3, power of 0.90 and alpha at 0.05 two‐tailed level significance. The least needed sample size was 196. To compensate for possible response rate, 240 HCPs were approached and 206 returned the completed survey with a response rate of 86%. The criteria for inclusion were any physician or nurse working in a general hospital in Jordan and able to comprehend Arabic.

### 2.2 Procedure

Approvals for conducting the study were obtained from the institution review committees at the authors' academic institution (Ref.: 10/124/2019). Data were collected using an electronic (online) format of surveys. The study, its purpose and significance of the research were announced via networking and social media. Information relating to the title, confidentiality, information privacy and anonymity of the survey was also included. Respondees who agreed to participate were provided with the link to the online survey, and the contact information of the researchers was also shared in case participants wished to make inquiries or ask related questions. A digital consent form was enclosed in the online survey which the HCP had to sign in order to be linked to the original surveys. Collected electronic information was saved to a password‐secured computer.

### 2.3 Study instruments

The participants reported their demographic data (age, gender, marital status, educational level, specialization, years of experience, previous training and education in mental health), in addition to their knowledge, attitude and behaviour.

To measure the HCPs' knowledge about mental illness, the mental health knowledge Schedule (MAKS) was administered (Evans‐Lacko et al., 2010). The MAKS is a 12‐item tool. The first 6 items measure stigma‐related mental health knowledge on a 5‐point Likert scale ranging from 1 = totally disagree to 5 = totally agree. The other 6 items (7–12) assess opinions about which conditions are types of mental health problems, measured on 3‐point responses. The total score is the sum of the responses to the first 6 items and ranges from 6 to 30 to which, a higher score indicates more knowledge. The MAKS is of a good test–retest reliability evidence 0.71 (Evans‐Lacko et al., 2010).

The Mental Illness: Clinicians' Attitudes‐version 4 (MICA v4; Gabbidon et al., 2013) was used to measure the attitudes of HCPs towards clients with mental illness. It is a 16‐item scale on a 6‐point Likert scale ranging from 1 = totally agree to 6 = totally disagree. Scores range from 16 to 96; lower score means less stigma. The MICA v4 was found to have good internal consistency (α = 0.72) and item‐total correlations (Gabbidon et al., 2013).

The Reported and Intended Behaviour Scale (RIBS) is used to measure the reported and intended behaviours of HCPs towards clients with mental illness (Evans‐Lacko et al., 2011). RIBS examines four contexts of intended and reported behaviour, namely those living with, working with, living nearby and having a relationship with a person suffering mental health issues. RIBS is an 8‐item survey. In the first four items, the prevalence of the four previously mentioned contexts is assessed, while the second four items examine the intended behaviour in the same contexts. It is important to note that because the respondents' experience of the behaviours in items 1 to 4 may vary the data is not included in the final score and is instead used only for measuring prevalence (Evans‐Lacko et al., 2011). Items 5–8 were measured on a 5‐point Likert scale. Total score ranges from 4 to 20; a higher score indicates greater willingness to contact people with mental illness. The RIBS overall test–retest reliability was 0.75 (Evans‐Lacko et al., 2011). For this study, the mentioned stigma‐components scales have been previously tested and evaluated and the Arabic versions by Dalky et al. (2020) were used.

### 2.4 Data analysis

Data analysis was conducted using the IBM‐SPSS 25. Descriptive statistics was carried out initially before conducting the main analysis. The variables (MICA v4, MAKS and RIBS) were assessed for normality, sample mean and standard deviation. To assess the association between variables, including characteristics relating to demographics, Pearson's correlations were conducted.

A series of t‐tests for two independent samples and ANOVA were conducted to detect the differences in study variables (knowledge, attitudes and behaviours) based on participants' characteristics. Further, regression analysis was conducted to identify factors that predict stigma components in this study. Significance level was set at 0.05.

## 3 RESULTS

The participant's mean age was 31.08 (SD = 6.70, Range = 21–64), and 58.7% were male. On average, the participants have 7.6 years of experience; most had less than four years' experience (41.3%). More than two‐thirds of the participants were nurses (77.2%), from public sectors (46.1%), and the psychiatry department (28%) (Table 1).

**TABLE 1:** Sociodemographic characteristics of participants (*N* = 206).

Variable	*N* (%)	Mean (SD)
Age (mean, SD)		31.08 (6.70)
Year of experience		7.67 (6.36)
Year of experience		
1–4	85 (41.3)	
5–9	55 (26.7)	
10–15	45 (21.8%)	
16–38	21 (10.2)	
Health sectors		
Public	71 (34.5)	
Private	95 (46.1)	
University	20 (9.7)	
Military	20 (9.7)	
Health professional		
Nurses	159 (77.2)	
Physician	47 (22.8)	
Gender		
Male	121 (58.7)	
Female	85 (41.3)	
Marital status		
Single	83 (40.3)	
Married	118 (57.3)	
Widowed/Divorced	5 (2.5)	
Educational level		
Diploma	11 (5.3)	
Bachelor	114 (69.6)	
Postgraduate	41 (24.7)	
Specialty		
Paediatric	10 (4.9)	
Medical surgical	55 (26.7)	
OPD	14 (6.8)	
Psychology	58 (28.2)	
Emergency	39 (18.9)	
Operation	3 (1.5)	
ICU	27 (13.1)	
Training in psychiatry		
Yes	153 (74.3)	
No	53 (25.7)	

### 3.1 Social cognitive components of stigma

#### 3.1.1 Knowledge: MAKS

Participants' knowledge about stigma was moderate, with an average score of 42.9 (± 4.78), indicating a good understanding of stigma‐related mental health. The lowest score was reported concerning grieves and stress, 2.47 (± 1.3) and stress 2.11 (± 1.22) respectively (Table 2).

**TABLE 2:** Participants' knowledge about stigma using MAKS (*N* = 206).

Item	*M* (SD)
1. Most people with mental health problems want to have paid employment	3.38 (1.09)
2. If a friend had a mental health problem, I know what advice to give them to get professional help	3.78 (1.05)
3.Medication can be an effective treatment for people with mental health problems	3.64 (1.24)
4. Psychotherapy (e.g., talking therapy or counselling) can be an effective treatment for people with mental health problems	3.92 (1.04)
5. People with severe mental health problems can fully recover	3.23 (1.21)
6. Most people with mental health problems go to a healthcare professional to get help (reversed)	2.89 (1.23)
7. Schizophrenia	4.66 (0.73)
8. Bipolar disorder (manic depression)	4.54 (0.93)
9. Depression	4.36 (1.03)
10. Drug addiction	3.94 (1.2)
11. Grief (reversed)	2.47 (1.3)
12. Stress (reversed)	2.11 (1.22)

#### 3.1.2 Attitude: MIKA

Participants revealed moderate negative attitude towards the people with mental illnesses with an average score of 53.79 (±9.15) (Table 3) and a range between (21 and 70). A high negative attitude (Mean > 3.5) was indicated in 8 out of 16 items of the survey. The highest negativity was reported in item “Health/social care staff know more about the lives of people treated for a mental illness than do family members and friends” (means score 4.69, ±1.21). Another high negativity score was reported in item “Being a health/social care professional in the area of mental health is not like being a real health/social care professional” (means score 4.27, ±1.34). On the other hand, least negativity score was reported in item “It is important that any health/social care professional supporting a person with mental illness also ensures that their physical health is assessed” (Mean score 2.09, ±1.24) (Table 3).

**TABLE 3:** Participants' attitudes towards stigma (*N* = 206).

Item	Mean (SD)
It is important that any health/social care professional supporting a person with mental illness also ensures that their physical health is assessed	2.09 (1.24)
If a senior colleague instructed me to treat people with mental illness in a disrespectful manner, I would not follow their instructions	2.22 (1.61)
Working in the mental health field is just as respectable as other fields of health and social care	2.23 (1.38)
If a colleague told me they had a mental illness, I would still want to work with them	2.50 (1.29)
I feel as comfortable talking to a person with mental illness as I do talking to a person with physical illness	2.75 (1.37)
I would use the terms “crazy,” “nutter,” “mad,” etc. to describe to colleagues people with mental illness that I have seen in my work	2.82 (1.7)
If a person with a mental illness complained of physical symptoms (such as chest pain), I would attribute it to their mental illness	3.19 (1.57)
The public does not need to be protected from people with mental illness	3.20 (1.44)
People with severe mental illness can never recover enough to have a good quality of life	3.74 (1.41)
I just learn about mental health when I have to, and would not bother reading additional material on it	3.87 (1.6)
General practitioners should not be expected to complete a thorough assessment for people with psychiatric symptoms because they can be referred to a psychiatrist	3.89 (1.67)
If I had a mental illness, I would never admit this to any of my friends because I would fear being treated differently	4.0 (1.5)
If I had a mental illness, I would never admit this to my colleagues for fear of being treated differently	4.02 (1.5)
People with mental illness are dangerous more often than not	4.25 (1.35)
Being a health/social care professional in the area of mental health is not like being a real health/social care professional	4.27 (1.34)
Health/social care staff know more about the lives of people treated for a mental illness than do family members and friends	4.69 (1.21)

#### 3.1.3 Behaviours: RIBS

The average score of the behaviour scale was 13.91 (±3.48), which indicated a moderate or not greater interest to deal with people with mental issues. This average score in behaviour could be related to the fact that half of the participants (48.5%) are currently working with such patients, and 48 (40%) of participants live with relatives with mental illness.

### 3.2 Bivariate analysis

The bivariate correlation revealed no significant correlation between stigma and age. However, negative significant correlation was found between HCPs knowledge and attitude indicating that HCPs with more knowledge were significantly have more positive attitude (lower average score) towards those suffering from the illness. A more significant correlation was found between HCPs' knowledge and behaviour. The HCPs who had more knowledge were holding more interest and willingness towards dealing with persons with mental illness.

Further, t‐tests and ANOVA were conducted to assess the differences in perceived stigma based on participants' characteristics (Table 4). Participants with 5 to 9 years of experience significantly had more negative attitudes than other experience categories (*p* = 0.003). Participants with 10 to 15 years of experience had a more significant interest in mentally ill people. Also, a significant difference in PHCs knowledge and attitude was revealed based on specialty. As such, participants with prior psychiatry training had a significantly higher interest score than those with no training (*p* < 0.001). No other significant differences were revealed (Table 4).

**TABLE 4:** Univariate analysis of the association with stigma components and study variables.

Variable	Knowledge	*p*‐vale	Attitude	*p*‐vale	Behaviour	*p*‐vale
Age	*r* = 0.016	0.82	*r* = 0.038	0.589	*r* = 0.026	0.711
Year of experience						
1–4	43.4(4.2)	0.123	52.4(9.9)	0.003	14.16(3.3)	0.001
5–9	41.78(5.4)		57.5(7.6)		12.63(3.3)	
10–15	43.68(4.6)		51.5(9.1)		15.33(3.5)	
16–38	42.14(4.6)		53.2(6.5)		13.19(3.2)	
Health sectors						
Public	43.56(4.8)	0.003	53.2(9.2)	0.855	13.98(3.3)	0.696
Private	41.69(4.8)		54.3(9.9)		14.06(3.5)	
University	45.35(3.9)		52.9(7.1)		13.05(3.1)	
Military	43.85(3.2)		53.3(6.7)		13.8(4.1)	
Marital status						
Single	43.07(4.5)	0.928	53.1(9.7)	0.493	14.06(3.1)	0.29
Married	42.82(4.9)		54.2(8.8)		13.91(3.6)	
Widowed	43.0(0)		56.5(0.7)		9.5(3.5)	
Divorced	41.33(8.0)		47.3(4.0)		12.66(5.8)	
Educational level						
Diploma	43.81(4.7)	0.544	54.9(7.7)	0.249	15.0(3.7)	0.641
Bachelor	42.58(4.7)		54.4(9.2)		13.79(3.4)	
Postgraduate	43.61(4.9)		51.2(9.4)		14.17(3.5)	
Specialty						
Paediatric	44.4(5.9)	<0.001	48.8(7.5)	0.001	14.5(2.6)	0.634
Medical surgical	43.83(4.4)		52.7(9.2)		13.67(3.7)	
OPD	45.78(3.2)		53.5(8.0)		13.07(4.6)	
Psychology	40.15(4.6)		58.06(8.6)		14.55(3.1)	
Emergency	43.53(4.5)		51.7(9.4)		13.94(3.3)	
Operation	44.0(3.6)		57.0(2.6)		13.0(5.5)	
ICU	43.81(4.2)		50.8(8.1)		13.29(3.3)	
Training in psychology						
Yes	42.9 (4.8)	0.977	53.6 (9.5)	0.826	14.4 (3.9)	<0.001
No	42.8 (4.7)		53.9 (7.8)		12.3 (4.8)	
Health professional						
Nurses	42.68(4.8)	0.231	53.6(9.3)	0.814	13.84(3.4)	0.632
Physician	43.63(4.5)		54.0(8.5)		14.12(3.7)	
Gender						
Male	42.82(4.8)	0.785	54.1(8.8)	0.384	14.28(3.5)	0.064
Female	43.01(4.7)		53.0(9.5)		13.37(3.3)	

## 4 DISCUSSION

Stigma is still a major barrier towards seeking and utilizing healthcare services, in particular concerning mental illness and associated patients. This study emphasized the perception of stigma elements (knowledge, attitudes, and behaviours) among hospital‐working HCPs. In this study, the high level of knowledge could be related to the fact that 74% of the participants reported having prior psychiatric training. Such training probably provided HCPs with up‐to‐date knowledge regarding mental health care leading to higher level of knowledge associated with mental illness. Yet, the results showed that HCPs moderate level of knowledge about mental illness would not hinder their negative attitudes and their willingness to contact with people with mental illness. The negative attitudes in this study indicated that HCPs do stigmatize the illness despite of their “modest” adequate professional and officially received knowledge or psychiatry training. Nevertheless, they keep their professional manners by trying to adopt positive behaviour towards people with mental illness as shown in the RIBS score levels. Such a contradicted reports can be explained in terms of lack of experience working with patients with mental illness and lack of adequate “specialized” psychiatry training in various mental health facilities spread across the healthcare sector in Jordan. A note of worthy to mention here is that in Jordan, nursing and medical education limit training of students to one single course that might not be enough to establish contact and communication with patients for future practice. It has been reported that nurses who had contact with patients with mental illnesses and who had been trained sufficiently in mental health and psychosocial support had more positive attitudes towards the illness itself and the related patients (Bjorkman et al., 2018; Stuhlmiller & Tolchard, 2019). To this, it is highly recommended to incorporate such modifications in the nursing and medical education curricula to extend duration of psychiatry training to better enhance contact and improve attitudes towards mental illness and patients with mental illness. Furthermore, Jordan like other neighbourhood countries is known to have limited number of mental health facilities and low mental health to patients' ratio (WHO, 2010; 2013). Stigma has reported as one related factor that relate to the low numbers of specialized HCPs in mental healthcare sector. This ultimately would have affected the quality outcome of mental healthcare services leading to less visibility of mental health specialty to public and other healthcare professionals (Dalky et al., 2020).

Indeed, more is needed concerning attitudes towards mental illness and overcoming associated mental health stigma among HCPs. This is a more specialized topic that requires further involvement of HCPs in general healthcare settings while caring for patients with mental illness and institutional efforts that need to be geared towards combating the three elements of stigma associated with mental illness. For the knowledge element, the academic preparation at the college level is not sufficient to combat stigma as much as increasing theoretical knowledge about mental illness. There should be more emphasis on rights of patients with mental illness and special topics related to increasing public awareness to the needs of patients with mental illness and other aspects of mental health care. For the attitude and behaviour elements; as guided by the Thornicroft model, positive behaviours reported by nurses or physicians infer by their commitment to professional manners in providing care and managing needs of patients with mental illness (Thornicroft et al, 2007; Hamilton et al, 2014). Such positive behaviours would enable the development of positive attitudes due to persistent contact with patients with mental illnesses. This is one novel finding of this study and adds to the body of knowledge and our understanding of underlying reasons for negative attitudes among HCPs. To assure these at a wider range, public base awareness campaigns to fight stigma associated with mental illness would help in eradicating associated stigma, increase knowledge, promote more positive attitudes and thus assure positive reported intended behaviours among different sectors of the general publication including HCPs working in general hospital settings.

One form of stigma that we have found in this study is related to fears to confess having mental illness to their peers and colleagues. Due to the stigma and negative attitudes, those with mental illness believe that disclosing their issues will result in differential treatment, loss of privileges, loss of responsibilities and even their job. These fears could be related to employment restricted context and difficulties related to management and treatment of mental illness which could influence their opportunities or career development. This is in consistent with other studies that addressed public concerns to disclose their mental illness fearing stigma (Chen et al., 2020; Hudson et al., 2021). HCPs do believe that persons suffering from mental illnesses are often dangerous and HCPs working with such patients do not have the same personality traits as other healthcare professionals. This may increase concerns among HCPs towards willingness to specialize in mental health or working directly with patients with mental illness. Another possible factor to consider for future nursing or medical graduates and their choice to practice or do their residency programme on.

As indicated by the high scores on the MICA scale, HCPs displayed negative attitudes towards mental illness. Similar negative attitudes were reported in a previous Jordanian study among nurses and physicians working in the community outpatient healthcare settings (Dalky et al., 2020). Further, the meta‐analysis report by Giandinoto et al. (2018) on mental health attitudes among general hospital staff concluded the existence of high levels of stigma due to inadequate mental health care, lack of skills, poor knowledge and lack of resources. Indeed, earlier intervention studies directed towards stigma reduction concluded that stigma related to mental health issues could be reduced entirely by providing specialized training programmes that are focused on enhancing knowledge, attitudes and behaviours successfully (Giandinoto et al., 2018; Li et al., 2014). In other studies, knowledge linked to mental health care has been combined with behaviour and attitudes, and progress has been achieved in determining the change in knowledge, attitude, and behaviour through training interventions (Petersen et al., 2016; Thornicroft et al., 2016).

Regarding experience, HCPs with longer years of work experience showed significantly lower levels of negative attitudes and higher frequency of positive behaviours towards mental illness compared to others with less experience. On the contrary, a systematic review concluded that HCPs with more years of experience showed higher negative attitudes towards mental illness in comparison to less experienced colleagues and were more pessimistic about the capability of people with mental illness to adhere to treatment protocol and recovery (Vistorte et al., 2018). Possible explanations to these contradicted results would be the cultural or contextual factors involved in the work settings in which those HCPs are currently working at. A suggestion for replicated studies in various context of mental healthcare stings to be conducted to better investigate factors associated with stigma perception among HCPs working in general healthcare settings or specialized mental health institutions.

This study has several strengths as it broadens the understanding of associative stigma among HCPs and the related factors; yet, it has few limitations. First, the utilization of cross‐sectional design study would hinder the causal relationship between stigma elements and participants’ demographical factors. Further, the use of convenient sampling technique in recruiting study participants would limit generalizability of the study findings, therefore; results in this current study should be considered with caution. Lastly, the online self‐report survey tool would carry the risk of participants answered with social favourable responses and thus raises the possibility of biased “social desirable answers.” This is a possible explanation for the contradicted results regarding the HCPs' high level of knowledge from previous psychiatry training and how it could predict their intended behaviours and their attitudes; which initially turned to be low.

## 5 CONCLUSION

This study evaluated stigma perception among hospital professionals. The results highlighted that HCP reported varied attitudes towards the mentally ill which could be due to prior special knowledge training. This knowledge was predicted to result in positive attitudes; yet in this study, the majority of HCPs are found to have negative attitudes which also reflect on their intended behaviours. This negativism raises the need to call for more specialized awareness programmes to tackle stigma and its components pertaining to mental illnesses. Doing so would likely assure higher standards of practice and proper quality of care provided for people with such problems.

## AUTHOR CONTRIBUTIONS

Conceptualization, methodology and data collection: Heyam F. Dalky, Ayman M. Hamdan‐Mansour, Ala'a F. Dalky, Malek Alnajar, Mohammad Al‐Ma'ani, and Naser Mahmoud. Data analysis and visualization: Malek Alnajar and Sultan Mosleh. Writing – original draft, Heyam F. Dalky, Mohammad Al‐Ma'ani, Naser Mahmoud, Sultan Mosleh, and Ayman M. Hamdan‐Mansour. Supervision and Project Administration, Heyam F. Dalky and Malek Alnajar. Writing – review and editing: Heyam F. Dalky and Ayman M. Hamdan‐Mansour.

## FUNDING INFORMATION

No external funding was required to support this work. The study method, writing of the report and the decision to submit for publication were made by the authors. The contributing members were not paid for their efforts.

## CONFLICT OF INTEREST STATEMENT

The authors declare that they have no known competing financial interests or personal relationships that could have appeared to influence the work reported in this paper.

## ETHICS STATEMENT

Approvals for conducting the study were obtained from the institution review committees at Jordan University of Science & Technology (Ref.: 10/124/2019).

## CONSENT FOR PUBLICATION STATEMENT

Consent was obtained from each participant prior to enrollment in the study stating data to be used for research publication purposes only.

## Data Availability

Data available on request due to privacy/ethical restrictions.

## References

[nop21953-bib-0001] Al Ali, N. M. , Alqurneh, M. K. , Dalky, H. , & Al‐Omari, H. (2017). Factors affecting help‐seeking attitudes regarding mental health services among attendance of primary health care centers in Jordan. International Journal of Mental Health, 46(1), 38–51.

[nop21953-bib-0002] Angermeyer, M. C. , & Dietrich, S. (2006). Public beliefs about and attitudes towards people with mental illness: A review of population studies. Acta Psychiatrica Scandinavica, 113(3), 163–179. 16466402 10.1111/j.1600-0447.2005.00699.x

[nop21953-bib-0003] Bjorkman, A. , Andersson, K. , Bergström, J. , & Salzmann‐Erikson, M. (2018). Increased mental illness and the challenges this brings for district nurses in primary care settings. Issues in Mental Health Nursing, 39(12), 1023–1030. 10.1080/01612840.2018.1522399 30624130

[nop21953-bib-0004] Chen, S. X. , Mak, W. W. , & Lam, B. C. (2020). Is it cultural context or cultural value? Unpackaging cultural influences on stigma toward mental illness and barrier to help‐seeking. Social Psychological and Personality Science, 11(7), 1022–1031. 10.1177/1948550619897482

[nop21953-bib-0005] Corrigan, P. W. , Watson, A. C. , & Barr, L. (2006). The self‐stigma of mental illness: Implications for self‐esteem and self‐efficacy. Journal of Social and Clinical Psychology, 25(8), 875–884. 10.1521/jscp.2006.25.8.875

[nop21953-bib-0006] Crocker, J. , & Lutsky, N. (1986). Stigma and the dynamics of social cognition. In Ainlay, S.C. , Becker, G. , Coleman, L.M. (eds) The Dilemma of Difference. Perspectives in Social Psychology. Boston, MA: Springer. doi: 10.1007/978-1-4684-7568-5_6

[nop21953-bib-0007] Cuschieri, S. (2019). The STROBE guidelines. Saudi Journal of Anesthesia, 13(Suppl. 1), S31–S34. 30930717 10.4103/sja.SJA_543_18 PMC6398292

[nop21953-bib-0008] Dalky, H. F. (2012). Arabic translation and cultural adaptation of the stigma‐devaluation scale in Jordan. Journal of Mental Health, 21, 72–82. 10.3109/09638237.2011.629238 22257132

[nop21953-bib-0009] Dalky, H. F. , Abu‐Hassan, H. H. , Dalky, A. F. , & Al‐Delaimy, W. (2020). Assessment of mental health stigma components of mental health knowledge, attitudes and behaviors among Jordanian healthcare providers. Community Mental Health Journal, 56(3), 524–531. 10.1007/s10597-019-00509-2 31760548 PMC10923396

[nop21953-bib-0010] Dalky, H. F. , Qandil, A. M. , Natour, A. S. , & Menninger, J. (2017). Quality of life, stigma, and burden perception among family caregivers and patients with psychiatric illnesses in Jordan. Community Mental Health Journal, 53, 266–274. 10.1007/s10597-016-0028-0 27272515

[nop21953-bib-0011] Evans‐Lacko, S. , Little, K. , Meltzer, H. , Rose, D. , Rhydderch, D. , Henderson, C. , & Thornicroft, G. (2010). Development and psychometric properties of the mental health knowledge schedule. Canadian Journal of Psychiatry, 55(7), 440–448. 10.1177/070674371005500707 20704771

[nop21953-bib-0012] Evans‐Lacko, S. , Rose, D. , Little, K. , Flach, C. , Rhydderch, D. , Henderson, C. , & Thornicroft, G. (2011). Development and psychometric properties of the reported and intended behaviour scale (RIBS): A stigma‐related behaviour measure. Epidemiology and Psychiatric Sciences, 20(3), 263–271. 10.1017/s2045796011000308 21922969

[nop21953-bib-0013] Gabbidon, J. , Clement, S. , Nieuwenhuizen, A. , Kassam, A. , Brohan, E. , Norman, I. , et al. (2013). Mental illness: Clinicians' attitudes (MICA) scale: Psychometric properties of a version for healthcare students and professionals. Psychiatric Research, 206, 81–87. 10.1016/j.psychres.2012.09.028 23084597

[nop21953-bib-0014] Giandinoto, J. A. , & Edward, K. L. (2015). The phenomenon of co‐morbid physical and mental illness in acute medical care: The lived experience of Australian health professionals. BMC Research Notes, 8, 1–9. 26148864 10.1186/s13104-015-1264-z PMC4494698

[nop21953-bib-0015] Giandinoto, J. A. , Stephenson, J. , & Edward, K. L. (2018). General hospital health professionals' attitudes and perceived dangerousness towards patients with comorbid mental and physical health conditions: Systematic review and meta‐analysis. International Journal of Mental Health Nursing, 27(3), 942–955. 10.1111/inm.12433 29399940

[nop21953-bib-0016] Goffman, E. (1963). stigma. notes on the management of spoiled identity. Penguin Books.

[nop21953-bib-0017] Gronholm, P. C. , Henderson, C. , Deb, T. , & Thornicroft, G. (2017). Interventions to reduce discrimination and stigma: The state of the art. Social Psychiatry and Psychiatric Epidemiology, 52, 249–258. 28144713 10.1007/s00127-017-1341-9 PMC5344948

[nop21953-bib-0018] Hamdan‐Mansour, A. M. , & Wardam, L. A. (2009). Attitudes of Jordanian mental health nurses toward mental illness and patients with mental illness. Issues in Mental Health Nursing, 30(11), 705–711. 19874099

[nop21953-bib-0019] Hamilton, S. , Lewis‐Holmes, E. , Pinfold, V. , Henderson, C. , Rose, D. , & Thornicroft, G. (2014). Discrimination against people with a mental health diagnosis: Qualitative analysis of reported experiences. Journal of Mental Health, 23, 88–93. 10.3109/09638237.2014.880408 24660972

[nop21953-bib-0020] Hudson, E. , Arnaert, A. , & Lavoie‐Tremblay, M. (2021). Healthcare professional disclosure of mental illness in the workplace: A rapid scoping review. Journal of Mental Health, 1–13. 10.1080/09638237.2021.1979485 34582294

[nop21953-bib-0021] Li, J. , Thornicroft, G. , & Huang, Y. (2014). Levels of stigma among community mental health staff in Guangzhou, China. BMC Psychiatry, 14, 231. 10.1186/s12888-014-0231-x 25115221 PMC4149249

[nop21953-bib-0022] Link, B. G. , Yang, L. H. , Phelan, J. C. , & Collins, P. Y. (2004). Measuring mental illness stigma. Schizophrenia Bulletin, 30(3), 511–541. 15631243 10.1093/oxfordjournals.schbul.a007098

[nop21953-bib-0023] Livingston, J. D. , & Boyd, J. E. (2010). Correlates and consequences of internalized stigma for people living with mental illness: A systematic review and meta‐analysis. Social Science & Medicine, 71(12), 2150–2161. 21051128 10.1016/j.socscimed.2010.09.030

[nop21953-bib-0024] Petersen, I. , Evans‐Lacko, S. , Semrau, M. , Barry, M. M. , Chisholm, D. , Gronholm, P. , Egbe, C. O. , & Thornicroft, G. (2016). Promotion, prevention and protection: Interventions at the population‐ and community‐levels for mental, neurological and substance use disorders in low‐ and middle‐income countries. International Journal of Mental Health Systems, 10, 30. 10.1186/s13033-016-0060-z 27069506 PMC4827227

[nop21953-bib-0025] Rayan, A. , & Aldaieflih, M. T. (2019). Public stigma toward mental illness and its correlates among patients diagnosed with schizophrenia. Contemporary Nurse, 55(6), 522–532. 31535959 10.1080/10376178.2019.1670706

[nop21953-bib-0026] Reavley, N. J. , Mackinnon, A. J. , Morgan, A. J. , & Jorm, A. F. (2014). Stigmatizing attitudes towards people with mental disorders: A comparison of Australian health professionals with the general community. Australian & New Zealand Journal of Psychiatry, 48(5), 433–441. 10.1177/0004867413500351 23943633

[nop21953-bib-0027] Stuber, J. P. , Rocha, A. , Christian, A. , & Link, B. G. (2014). Conceptions of mental illness: Attitudes of mental health professionals and the general public. Psychiatric Services, 65(4), 490–497. 24430508 10.1176/appi.ps.201300136 PMC4028068

[nop21953-bib-0028] Stuhlmiller, C. , & Tolchard, B. (2019). Understanding the impact of mental health placements on student nurses' attitudes towards mental illness. Nurse Education in Practice, 34, 25–30. 10.1016/j.nepr.2018.06.004 30408765

[nop21953-bib-0029] Thornicroft, G. , Mehta, N. , Clement, S. , Evans‐Lacko, S. , Doherty, M. , Rose, D. , Koschorke, M. , Shidhaye, R. , O'Reilly, C. , & Henderson, C. (2016). Evidence for effective interventions to reduce mental‐health‐related stigma and discrimination. The Lancet, 387(10023), 1123–1132. 10.1016/S0140-6736(15)00298-6 26410341

[nop21953-bib-0030] Thornicroft, G. , Rose, D. , Kassam, A. , & Sartorius, N. (2007). Stigma: Ignorance, prejudice or discrimination? The British Journal of Psychiatry: The Journal of Mental Science, 190, 192–193. 10.1192/bjp.bp.106.025791 17329736

[nop21953-bib-0031] Vistorte, A. , Ribeiro, W. S. , Jaen, D. , Jorge, M. R. , Evans‐Lacko, S. , & Mari, J. J. (2018). Stigmatizing attitudes of primary care professionals towards people with mental disorders: A systematic review. International Journal of Psychiatry in Medicine, 53(4), 317–338. 10.1177/0091217418778620 29807502

[nop21953-bib-0032] World Health Organization . (2010). Mental health gap action program (mhGAP): 2nd meeting of the mhGAP Forum. Retrieved November 9, 2019, from https://www.who.int/mental_health/mhgap/mhgap_forum_oct2010_annex_e.pdf

[nop21953-bib-0033] World Health Organization . (2013). Investing in mental health: evidence for action. Retrieved November 9, 2019, from https://www.who.int/mental_health/publications/financing/investing_in_mh_2013/en/

